# Feedback regulation in a stem cell model with acute myeloid leukaemia

**DOI:** 10.1186/s12918-018-0561-2

**Published:** 2018-04-24

**Authors:** Jianfeng Jiao, Min Luo, Ruiqi Wang

**Affiliations:** Department of Mathematics, Shanghai University, Shangda Road No.99, Shanghai, 200444 China

**Keywords:** feedback regulation, haematopoietic stem cells, acute myeloid leukaemia, Hill function, mathematical model

## Abstract

**Background:**

The haematopoietic lineages with leukaemia lineages are considered in this paper. In particular, we mainly consider that haematopoietic lineages are tightly controlled by negative feedback inhibition of end-product. Actually, leukemia has been found 100 years ago. Up to now, the exact mechanism is still unknown, and many factors are thought to be associated with the pathogenesis of leukemia. Nevertheless, it is very necessary to continue the profound study of the pathogenesis of leukemia. Here, we propose a new mathematical model which include some negative feedback inhibition from the terminally differentiated cells of haematopoietic lineages to the haematopoietic stem cells and haematopoietic progenitor cells in order to describe the regulatory mechanisms mentioned above by a set of ordinary differential equations. Afterwards, we carried out detailed dynamical bifurcation analysis of the model, and obtained some meaningful results.

**Results:**

In this work, we mainly perform the analysis of the mathematic model by bifurcation theory and numerical simulations. We have not only incorporated some new negative feedback mechanisms to the existing model, but also constructed our own model by using the modeling method of stem cell theory with probability method. Through a series of qualitative analysis and numerical simulations, we obtain that the weak negative feedback for differentiation probability is conducive to the cure of leukemia. However, with the strengthening of negative feedback, leukemia will be more difficult to be cured, and even induce death. In contrast, strong negative feedback for differentiation rate of progenitor cells can promote healthy haematopoiesis and suppress leukaemia.

**Conclusions:**

These results demonstrate that healthy progenitor cells are bestowed a competitive advantage over leukaemia stem cells. Weak *g*_1_, *g*_2_, and *h*_1_ enable the system stays in the healthy state. However, strong *h*_2_ can promote healthy haematopoiesis and suppress leukaemia.

## Background

**Fig. 1:**
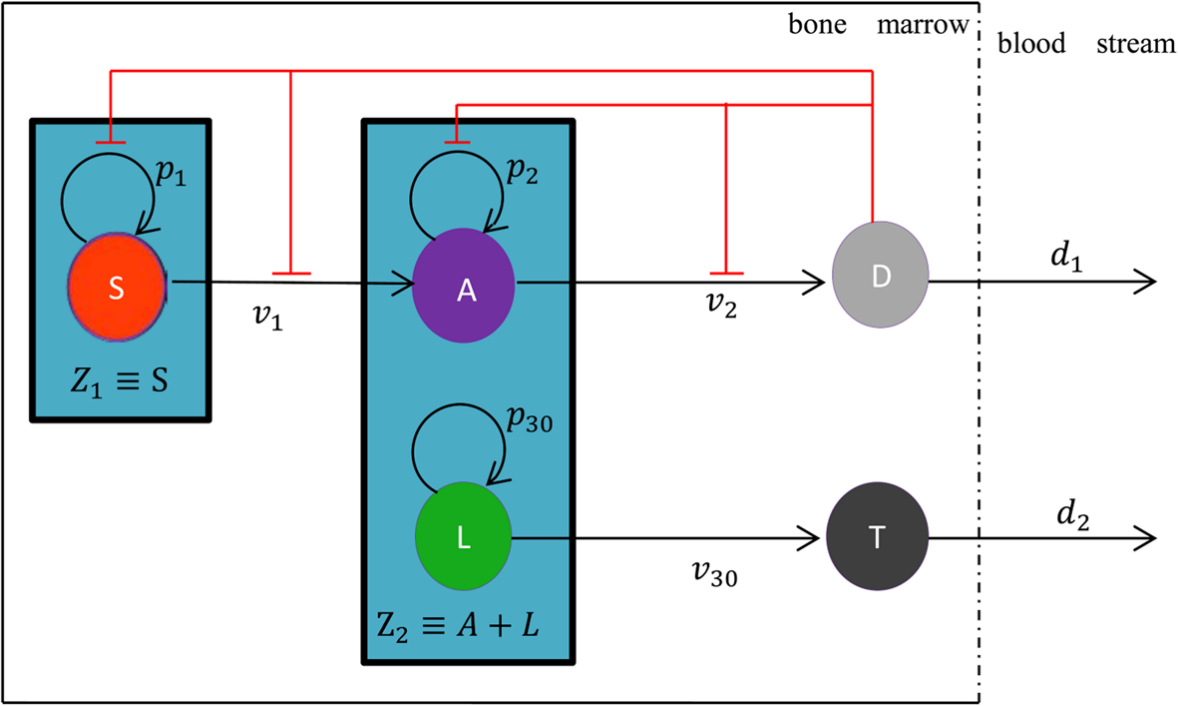
Schematic diagram of leukemogenesis and haematopoietic stem cell lineages. Upper row: haematopoietic linage. Lower row: leukaemia linage. The flat-head arrows extending from terminally differentiated cell (D) with red color indicates feedback inhibition to self-renewal of S and A (colour online)

Acute myeloid leukemia (AML) are a group of clonal diseases originating in the myeloid stem cells or myeloid progenitors. They are characterized by the accumulation of immature (blastic) myeloid cells in bone marrow, blood and less often in other areas and by a syndrome of bone marrow failure [1]. Bone marrow is the only hematopoietic site in adults, and in which there are many hematopoietic stem cells. Those stem cells belong to undifferentiated cells which are characterized both by multipotency (ability to produce many cell types) and their ability to maintain their own numbers through self-replication. Firstly, they can differentiate into oligopotent progenitors by regulation of many internal signals (the future direction of the cell differentiation has been determined at this moment), which then produce unipotent progenitors [2], e.g., the development of haematopoietic lineages shown in Fig. 1. However, pluripotent hematopoietic stem cell within the bone marrow is possible to form mutant cell, that is, leukemia cell due to micro-environmental change, genetic mutations or other factors.

**Fig. 2:**
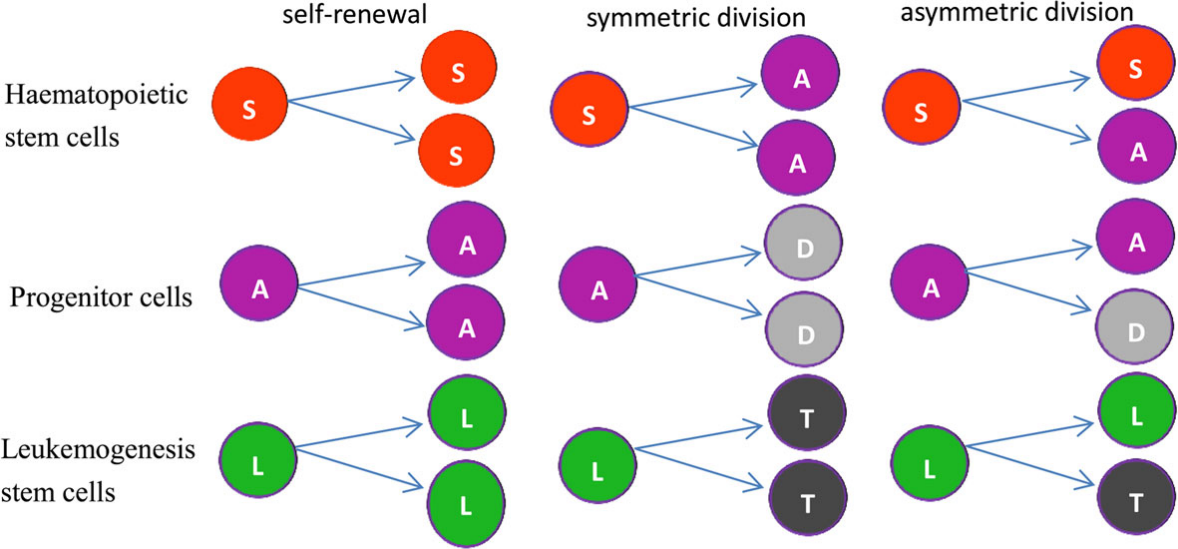
Division types of stem cells and progenitor cells. Red sphere labeled with (S) indicates haematopoietic stem cells, purple sphere (A) denotes progenitor cells, grey sphere (D) denotes terminally differentiated cells of S, green sphere (L) indicates leukaemia stem cells, and thicker grey sphere (T) indicates terminally differentiated cells of L (colour online)

On the one hand, according to their sources, leukemia cells can be divided into two categories: myeloid leukemia cells (which stem from hematopoietic stem cells or hematopoietic progenitors) and lymphoblastic leukemia cells (which stem from lymphoblastic progenitors). On the other hand, depending on the different rate of the development of leukemia, they can be divided into acute leukemia and chronic leukemia. In this paper, the acute myeloid leukemia (AML) is mainly considered. In addition, as shown in [3], cell division can be classified into three categories: self-renewal or proliferation, symmetric division, and asymmetric division. All these types of divisions for stem and progenitor cells are illustrated in Fig. 2. Although asymmetric cell division is possible, its introduction does not influence our results (please refer to literature [4] for the specific proof). So in our paper the case of asymmetric cell divisions will not be considered.

**Fig. 3:**
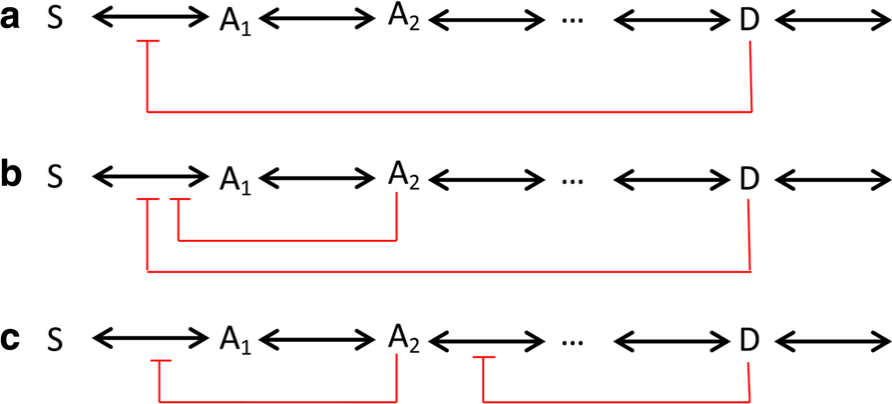
Strategies for regulation. **a** denotes the end-product inhibition regulatory scheme, **b** denotes a nest feedback scheme, and **c** denotes a sequential feedback scheme. Adapted from Figure 5.4 of [9] (colour online)

In the process of revealing the pathogenesis of leukemia, some studies found that leukaemia stem cells share more features with hematopoietic progenitor cells than hematopoietic stem cells [5–7]. Therefore, the competition between hematopoietic progenitor cells and leukaemia stem cells which leads to an increased cell death due to over-crowding in niche is considered. This type of competition is supported by experimental findings [8]. Here, the total niche size of the hematopoietic stem cells and the hematopoietic progenitors with leukaemia stem cells are represented as *Z*_1_=*S* and *Z*_2_=*A*+*L*, respectively. Up to now, different inhibition strategies have been used. To sum up, there are several types as shown in Fig. 3a denotes the end-product inhibition regulatory scheme, (*b*) denotes a nest feedback scheme, and (*c*) denotes a sequential feedback scheme. As shown in [9], though end-product inhibition has a defect which is prone to instability, it exhibits better performance than alternative feedback architectures, such as robustness to disturbances in demand for product *D*. Therefore, end-product inhibition strategy is adopted in our model instead of others.

Moreover, numerical simulations of the proposed model, validated by clinical observations of hematopoiesis after bone marrow transplantation [10–13], indicate that the regulation of self-renewal is a more efficient mechanism than the regulation of proliferation rates. Similar conclusions were drawn using the models of multistage cell lineages applied to regeneration and maintenance of the mouse olfactory epithelium [13, 14]. However, Lander et al. not only experimentally verified the existence of feedback loops which aimed at stem cells, but also verified the existence of feedback loops which aimed at transit-amplifying cells (progenitor cells) in [13]. What’s more, they have analyzed that double inhibition are better than single inhibition, that is, only feedback onto *p*_*i*_ hardly improves regeneration speed at all, but feedback onto both *p*_*i*_ and *v*_*i*_ (*i*=1,2) can produce rates of regeneration (the specific meaning of *p*_*i*_ and *v*_*i*_ will be explained below). Therefore, in this paper, we take double inhibition into account. In fact, we can assume that cytokine molecules inhibit the self-renewal probabilities of haematopoietic stem-cell and haematopoietic progenitor cell, so do proliferation rates in the present work.

The remainder of this paper is organized as follows. In the next subsection, we analyse the dynamical behavior of system (1)-(5) (which will be described later) by using bifurcation theory and numerical simulations to consider how progenitor cells interact and compete with the leukaemia stem cells in its surroundings. The paper ends with conclusions in the final section.

## Methods

### A competition model with endogenous negative feedback

**Fig. 4:**
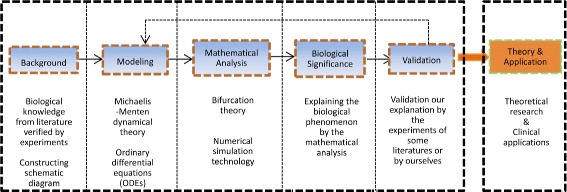
Flow diagram for the modeling and analytical method of this paper

Based on the literature [15], we know that ordinary differential equations can be applied to biochemical networks only under the following two key assumptions: one is the continuum hypothesis, and the other one is well-mixed assumption. The former allows us to measure species abundance as a continuously changing concentration rather than a discrete number of molecules, the latter provided that the reactants find one another immediately and equally, and the time scale of the process under investigation is longer than the time scale of diffusion of its components. Under those key assumptions we can solve many problems of life sciences by modelling. A flow diagram of the modeling process is summarized in Fig. 4.

In addition, we must understand other knowledge in order to develop a mathematical model. Since the English physiologist A.V. Hill proposed a function to fit the sigmoidal binding curve of oxygen for hemoglobin, it has been widely and successfully used to describe the ligand-receptor interactions in a variety of forms. Hence, we use Hill function to describe the negative feedback mechanism in our model. The following forms can be taken:

$$ p_{i}(D) = \frac{p_{i0}}{1+g_{i}D^{n}}, \quad v_{i}(D) = \frac{v_{i0}}{1+h_{i}D^{n}}\quad (i=1,2) $$

with the feedback coefficients *g*_*i*_≥0, *h*_*i*_≥0(*i*=1,2). *p*_*i*0_, *v*_*i*0_ are the maximal differentiation probability, where 0<*p*_*i*0_<1, *v*_*i*0_>0, (*i*=1,2,3), and *m*, *n* denote the Hill exponents. Obviously, *p*_1_(0)=*p*_10_, *p*_2_(0)=*p*_20_, and both feedbacks are decreasing functions of the number of differentiation cells, and converge to zero if it tends to infinity.

We describe the dynamics of the five species with the following ordinary differential equations. A schematic description of (1)-(5) is given in Fig. 1.

1 $$\begin{array}{@{}rcl@{}} \frac{dS}{dt}&=&\left[p_{1}(D)(K_{1}-Z_{1})-(1-p_{1}(D))\right]v_{1}(D)S,  \end{array} $$

2 $$\begin{array}{@{}rcl@{}} \frac{dA}{dt}&=&2(1-p_{1}(D))v_{1}(D)S+[p_{2}(D)(K_{2}-Z_{2}) \\ &&-(1-p_{2}(D))]v_{2}(D)A,  \end{array} $$

3 $$\begin{array}{@{}rcl@{}} \frac{dD}{dt}&=&2(1-p_{2}(D))v_{2}(D)A-d_{1}D,  \end{array} $$

4 $$\begin{array}{@{}rcl@{}} \frac{dL}{dt}&=&\left[p_{30}(K_{2}-Z_{2})-(1-p_{30})\right]v_{30}L,  \end{array} $$

5 $$\begin{array}{@{}rcl@{}} \frac{dT}{dt}&=&2(1-p_{30})v_{30}L-d_{2}T,  \end{array} $$

where *p*_*i*_(*D*) (*i*=1,2) and *p*_30_ denote the self-renewal probability of the haematopoietic stem cells, progenitor blood cells and leukaemia stem cells, respectively, while the corresponding divisive rate of them is represented as *v*_*i*_(*D*) (*i*=1,2) and *v*_30_. The parameters *d*_1_ and *d*_2_ denote the death rates of the differentiated blood cells *D* and *T*, respectively. *K*_1_ and *K*_2_ are the carrying capacities of the population sizes of cells within the bone marrow, and we scale the population sizes such that *K*_1_=*K*_2_=1 in our paper. Basal values and definitions of the model parameters are given in Table 1.

**Table 1:** Parameters set which describes system (1)-(5)

Parameter	Definition	Basal value	Reference
*p*_10_	The maximal differentiation probability of *S*	0.65	[4]
*p*_20_	The maximal differentiation probability of *A*	0.68	[4]
*p*_30_	The maximal differentiation probability of *L*	0.80	[4]
*v*_10_	The rate of haematopoietic stem cell division	0.50	[4]
*v*_20_	The rate of progenitor cell division	0.72	[4]
*v*_30_	The rate of leukemogenesis stem cell division	0.70	assume
*d*_1_	Rate constant for D cells undergo apoptosis or death	0.275	[7]
*d*_2_	Rate constant for T cells undergo apoptosis or death	0.30	[7]
*g*_1_	Feedback coefficient of *S* from *D*	0.03	[2]
*g*_2_	Feedback coefficient of *A* from *D*	0.025	[2]
*h*_1_	Feedback coefficient of *S* from *D*	0.2	assume
*h*_2_	Feedback coefficient of *A* from *D*	0.11	assume
*m*	Hill exponent of *p*_2_(*D*)	1	
*n*	Hill exponent of *p*_1_(*D*)	1	
*K*_1_	The carrying capacities of the compartment with *S*	1	[7]
*K*_2_	The carrying capacities of the compartment with *A* and *L*	1	[7]

## Results

### Steady state analysis

In fact, the system (1)-(5) has several steady states. However, according to the literature [10], only three types of steady state for a fixed set of parameters are considered from the perspective of biology: $P_{1}^{*}(0,0,0,L^{*},T^{*}), P_{2}^{*}(S^{*},A^{*},D^{*},0,0), P_{3}^{*}(S^{*},A^{*},D^{*},L^{*},T^{*}). P_{1}^{*}$ is a steady state of the form *S*^∗^=0,*A*^∗^=0,*D*^∗^=0 which is named purely leukemic steady state. In fact, this is an ideal state described in [10] and the abstract reference cannot be observed in reality since the organism dies in absence of health blood cells. Simultaneously, the corresponding case of *L*^∗^=0 and *T*^∗^=0, that is, $P_{2}^{*}$, is considered. As defined in [10], this case is referred to as the healthy steady state. It is also an abstract idealization, since in each organism mutations accumulate over lifetime due to replication errors. And $P_{3}^{*}$ is the steady state with coexisting healthy and leukaemia species.

For steady state $P_{1}^{*}$, only leukaemia lineages exist in the bone marrow microenvironment. From Eqs. 4 and (5), by letting *S*=*A*=*D*=0, we can obtain that $L^{*}=2-\frac {1}{p_{30}}$ and $T^{*}=-{\frac { 2\left (p_{30}-1\right) \left (2p_{{30}}-1 \right)v_{{30}} }{p_{{30}}d_{{2}}}}.$ The eigenvalues of the Jacobian matrix at $P_{1}^{*}$ are

$$\begin{aligned} \lambda_{1}=(2p_{10}-1)v_{10},\,\lambda_{2}=\frac{(p_{20}-p_{30})v_{20}}{p_{30}},\\ \lambda_{3}=-d_{1},\,\lambda_{4}=(1-2p_{30})v_{30},\,\lambda_{5}=-d_{2}. \end{aligned} $$

**Fig. 5:**
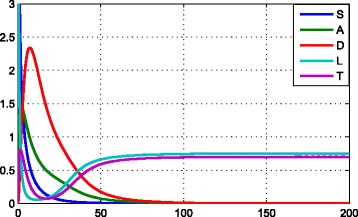
Establishment of a leukemic steady state and extinction of healthy cells. Parameters: In addition to *p*_10_, the values of other parameters please refer to Table 1. Here we choose *p*_10_=0.45. Initial conditions: S(0)=10, A(0)=0, D(0)=0, L(0)=10, T(0)=0 (colour online)

Therefore, $P_{1}^{*}$ is locally stable if and only if *p*_10_<0.5, *p*_20_<*p*_30_, and *p*_30_>0.5. From biological perspective, we can analyse that leukemic stem cells have enhanced self-renewal potential, but healthy stem cells have decreased self-renewal potential, and healthy progenitor cells have weak self-renewal potential compared to leukemic stem cells. This means that in the competition between healthy progenitor cells and leukemic stem cells, leukemic stem cells will be at a advantage. As a result, leukemia will be lasting though the divisive rate of leukemic stem cells is smaller than that of healthy cells (see Fig. 5).

For steady state $P_{2}^{*}$, only haematopoietic lineages exist in the bone marrow microenvironment. So by letting *L*=*T*=0, we obtain that $S^{*}=2-\frac {1}{p_{1}(D^{*})}$, $A^{*}=-\frac {d_{1}D^{*}}{2v_{20}(p_{2}(D^{*})-1)}$, and *D*^∗^ satisfies the following equation of six degree

6 $$ \begin{aligned} F(D) &:=a_{1}D^{*6}+a_{2}D^{*5}+a_{3}D^{*4}+a_{4}D^{*3}+a_{5}D^{*2} \\ &\quad +a_{6}D^{*1}+a_{0}=0, \end{aligned}  $$

where

$${\begin{aligned} a_{0}&=8\,v_{{10}}v_{{20}} \left(p_{{20}}-1 \right)^{2} \left(2\,p_{{10}}- 1 \right) \left(p_{{10}}-1 \right), \\ a_{1}&=p_{{20}}p_{{10}}d_{{1}}^{2}g_{{1}}g_{{2}}h_{{1}}h_{{2}}, \\ a_{2}&=d_{{1}}p_{{20}} \left(g_{{1}}g_{{2}}h_{{1}}+g_{{1}}g_{{2}}h_{{2}}+g_{{1}}h_{{1}}h_{{2}}+g_{{2}}h_{{1}}h_{{2}} \right) \\ & +2\,g_{{1}}g_{{2}}^{2}h_{{1}}v_{{20}}, \\ a_{3}&=p_{{10}}p_{{20}} \!\left(g_{{1}}g_{{2}}+g_{{1}}h_{{1}}+g_{{1}}h_{{2}}+g_{{2}}h_{{1}}+g_{{2}}h_{{2}}+\! h_{{1}}h_{{2}} \right)\! d_{{1}}^{2}\\ &\quad +2g_{{2}}p_{{10}}v_{{20}} \left(g_{{1}}g_{{2}}-3g_{{1}}h_{{1}}p_{{20}}+2\,g_{{1}}h_{{1}}+g_{{2}}h_{{1}} \right) d_{{1}} \\ &\quad +8g_{{1}}^{2}g_{{2}}^{2}v_{{10}}v_{{20}}, \\ a_{4}&=p_{{10}}p_{{20}} \left(g_{{1}}+g_{{2}}+h_{{1}}+h_{{2}} \right) d_{{1}}^{2} \\ &\quad -2\,p_{{10}}v_{{20}} \left(3 \,g_{{1}}g_{{2}}p_{{20}}-2\,g_{{1}}h_{{1}}p_{{20}}^{2} +3\,g_{{1}}h_{{1}}p_{{20}}\right.\\ &\quad \left. +3\,g_{{2}}h_{{1}}p_{{20}} -2\,g_{{1}}g_{{2}}-g_{{1}}h_{{1}}-g_{{2}}^{2}-2\,g_{{2}}h_{{1}} \right) d_{{1}} \\ &\quad -8\,g_{{1}}g_{{2}}v_{{10}}v_{{20}} \left(2\,g_{{1}}p_{{20}}+3\,g_{{2}}p_{{10}}-2\,g_{{1}}-2\,g_{{2}} \right), \\ a_{5}&=d_{{1}}^{2}p_{{10}}p_{{20}}+2\,p_{{10}}v_{{20}} \left(2\,g_{{1}}{p_{{20}}}^{2}+2\,h_{{1}}p_{{20}}^{2} \,-\, 3\,g_{{1}}p_{{20}} \right. \\ &\quad \left. -3\,g_{{2}}p_{{20}}-3\,h_{{1}}p_{{20}} + g_{{1}}+2\,g_{{2}}+h_{{1}} \right) d_{{1}} \\ &\quad +8\, v_{{10}}v_{{20}} \left(g_{{1}}^{2}p_{{20}}^{2}+6\,g_{{1}}g_{{2}}p_{{10}}p_{{20}}+2\,g_{{2}}^{2}p_{{10}}^{2}-2g_{{1}}^{2}p_{{20}}\right. \\ &\quad \left.-6\,g_{{1}}g_{{2}}p_{{10}} \,-\, 4\,g_{{1}}g_{{2}}p_{{20}} \,-\, 3\,g_{{2}}^{2} p_{{10}} \,+\, g_{{1}}^{2} \!+ 4\,g_{{1}}g_{{2}}+g_{{2}}^{2} \right), \\ a_{6}&=2\left(p_{{20}}-1 \right) v_{{20}} \left(p_{{10}} \left(2\,p_{{20}}-1 \right) d_{{1}} \,-\, 4\,v_{{10}} \left(3\,g_{{1}}p_{{10}}p_{{20}} \right.\right. \\ &\quad \left.\left. +4 \,g_{{2}}p_{{10}}^{2} \,-\, 3\,g_{{1}}p_{{10}} \,-\, 2\,g_{{1}}p_{{20}} \,-\, 6\,g_{{2}} p_{{10}}+2\,g_{{1}}+2\,g_{{2}} \right) \right). \end{aligned}} $$

Actually, due to the complexity of the solution of (6), the steady state behaviour of the steady state $P_{2}^{*}$ and $P_{3}^{*}$ analytically is impractical. In order to further analyse them, we appeal to analyse the model (1)-(5) using bifurcation theory and numerical technique in the following sections.

### The effect of negative feedback

Bifurcation is an universal phenomenon which means when the parameters pass through a critical value, the topological structure of the system will change. In this paper, system (1)-(5) undergoes a transcritical bifurcation at different equilibria. The stability of two different solutions is exchanged at the critical point of the bifurcation parameters.

**Fig. 6:**
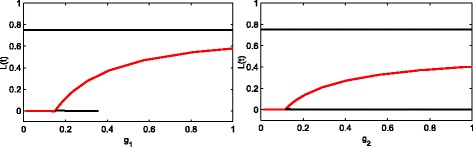
Single parameter bifurcation diagram of *g*_1_ and *g*_2_. The left diagram shows that there is a transition from healthy steady state to the coexisting steady state of the healthy and leukaemia species at *g*_1_=0.1499. Similarly, the right diagram indicates the same transition at *g*_2_=0.3869. The red solid line is a stable steady state, while the black solid line is an unstable steady state (colour online)

In Fig. 6, we draw the single parameter bifurcation diagram of *g*_1_ and *g*_2_, respectively. In this figure, the line with *L*(*t*)=0 denotes the healthy steady state, the line with *L*(*t*)=0.75 indicates purely leukemic steady state, and the composite steady state between them (the same as all of the following graphics). At the left of Fig. 6, when *g*_1_<0.1499, the inhibition from D is not so strong that the system will stay at the healthy steady state. Otherwise, in a large range of *g*_1_>0.1499, the system will stabilize at the coexisting steady state of the healthy and leukaemia species. When *g*_1_ is large enough, the system will be stable at the purely leukemic steady state which is not shown. Obviously, the disappearance of the healthy steady state (which is the black line in the left of the Fig. 6) when *g*_1_=0.3689 is found, which is actually a bifurcation point, but the steady state after bifurcation is not what we need from a biological perspective. The right figure is the single parameter bifurcation diagram of *g*_2_. Its analysis is similar to the left. Here we will not repeat it.

**Fig. 7:**
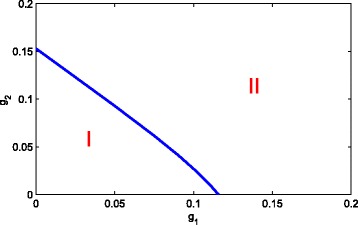
Bifurcation diagram in the parameter space of *g*_1_ and *g*_2_. When *g*_1_<0.115 or *g*_2_<0.152, they could co-compensate the inefficiency of each other in order to maintain in the state of health. However, when *g*_1_>0.115 or *g*_2_>0.152, they induce a state transition, that is, a stable composite state appear. The dynamics of the system can be divided into two classes: (I) healthy steady state and (II) the coexisting steady state of the healthy and leukaemia species (colour online)

**Fig. 8:**
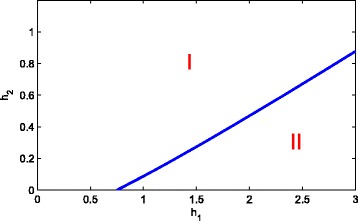
Bifurcation diagram in the parameter space of *h*_1_ and *h*_2_. When *h*_1_<0.3689 or *h*_2_>0.8861, they could co-compensate the inefficiency of each other in order to maintain in the state of health. However, when *h*_1_>0.3689 or *h*_2_<0.8861, they induce a state transition, that is, a stable composite state appear. The dynamics of the system can be divided into two classes: (I) healthy steady state and (II) the coexisting steady state of the healthy and leukaemia species (colour online)

In Fig. 7, one can see a bifurcation diagram in the parameter space of *g*_1_ and *g*_2_. In area I, the system will stay at healthy steady state. The negative feedback to the self-renewal of S and A could co-compensate the inefficiency of each other in order to maintain the healthy state. Afterwards, a state transition occurs with the increase of *g*_1_ and *g*_2_, that is, a stable composite state appear in area II. However, if *g*_1_ and *g*_2_ are large enough, both *p*_1_(*D*) and *p*_2_(*D*) approach zero. From system (1)-(5), we can only obtain a purely leukemic steady state and it is a stable state by simple computation. So, when the end-product inhibition is strong enough, it is disadvantage to cure the leukemia. This phenomenon is not difficult to observe from Fig. 6. In addition, from Fig. 6 one can find that *g*_1_ and *g*_2_ is synergistic, i.e, the inhibition from D to the differentiation probability of S and A is enhanced each other. The same phenomenon can be found in Fig. 8.

**Fig. 9:**
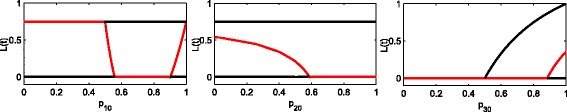
Single parameter bifurcation diagram of *p*_*i*0_ (i=1,2,3) (colour online)

**Fig. 10:**
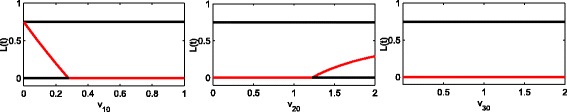
Single parameter bifurcation diagram of *v*_*i*0_ (i=1,2,3) (colour online)

In the next section, we will consider the transcritical bifurcation of the differentiation probability *p*_*i*0_ and the divisive rate *v*_*i*0_ (*i*=1,2,3) in Figs. 9 and 10, respectively.

### Competition between A and L

In this section, we will focus our attention on the regulation of the competition between the haematopoietic progenitor cells A and the leukaemia stem cells L. We want to understand how they interact and compete with each other in bone marrow microenvironment by our analysis. In Fig. 9, we perform bifurcation analysis with *p*_10_,*p*_20_, and *p*_30_ as bifurcation parameters, respectively. Firstly, in Fig. 9a, we found that the stable steady state can switch from purely leukemic steady state to the coexisting steady state when the parameter *p*_10_ passes through 0.5. Afterwards, the coexisting steady state can reach the healthy steady state as the parameter *p*_10_ reaches 0.5581. Finally, when *p*_10_=0.9019, the system will switch back to the coexisting steady state, and eventually when *p*_10_=1 to achieve purely leukemic steady state. When *p*_10_<0.5, as the analysis in section 3.1, the system will always be in the purely leukemia state with the condition that *p*_20_<*p*_30_ and *p*_30_>0.5. These phenomena can be explained from the biological perspective. Because the self-renewal ability of hematopoietic stem cells is weaker than that of differentiation, and the probability of self-renewal of A is less than that of L, few hematopoietic progenitor cells are produced, which leads to a disadvantage in the process of competition with leukemia stem cells. However, when 0.5<*p*_10_<0.5581, the situation has eased, the self-renewal ability of hematopoietic stem cells gradually strengthened, the number of progenitor cells A began to have a certain amount of accumulation which leads to our system stay in coexisting steady state. Afterwards, with the further enhancement of the self-renewal ability of hematopoietic stem cells, which leads to a greater advantage in the process of competition with leukemia stem cells, making the system will be in a healthy steady state. While the system will go back to the coexisting steady state because the self-renewal ability of hematopoietic stem cells is so strong that there is almost no hematopoietic progenitor cell formation.

In Fig. 9b, *p*_20_=0.5878 is a switching point where the system will be transformed from a coexisting steady state to a healthy steady state and remains in this state as the parameter *p*_20_ continues to increase. In fact, when *p*_20_<0.5878, the self-renewal ability of hematopoietic progenitor cells is not so powerful that they beat the leukaemia stem cells, therefore, the system stays in coexisting a steady state. However, when *p*_20_>0.5878, the situation was reversed, which leads to a large accumulation of progenitor cells A, which shows that the competition in the leukaemia stem cells is advantageous in the bone marrow micro-environment. In Fig. 9c, *p*_30_=0.5 is a point where purely leukemic steady state will appear, but it is not a switching point. The system is still stable in a healthy steady state until *p*_30_=0.8839 at which the stable steady state can switch from a healthy steady state to a coexisting steady state. Eventually, under the given values of the parameters, the system will be in the healthy steady state. While when the self-renewal ability of leukaemia stem cells become strong enough, a certain number of leukemia stem cells can be accumulated and the system will be in the coexisting state.

In Fig. 10, we have a similar description to Fig. 9. In Fig. 10a, the stability of different solutions is exchanged when *v*_10_=0.2791, that is, it is a transcritical bifurcation point where the system switches from a coexisting steady state to a healthy steady state. In Fig. 10b there is also a transcritical bifurcation at *v*_20_=1.224, but it switches from a healthy steady state to a coexisting steady state. While in Fig. 10c there is no state transition. In fact, this is consistent with Eq. 4 because the positive equilibrium is independent of *v*_30_.

**Fig. 11:**
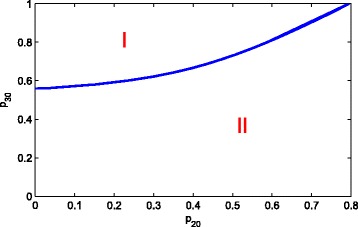
Bifurcation diagram in the parameter space of *p*_20_ and *p*_30_. When *p*_20_>0.7920 or *p*_30_<0.5587, the system maintains in the state of health. However, when *p*_20_<0.7920 or *p*_30_>0.5587, they induce a state transition, that is, a stable composite state appear. The dynamics of the system can be divided into two classes: (I) the coexisting steady state of the healthy and leukaemia species and (II) healthy steady state (colour online)

In Fig. 11, one can see a bifurcation diagram in the parameter space of *p*_20_ and *p*_30_. When *p*_20_>0.792 or *p*_30_<0.5587, they could co-compensate the inefficiency of each other in order to maintain the heathy state. However, when *p*_20_<0.7390 or *p*_30_>0.5587, they induce a state transition, that is, a stable composite state appear. Eventually, area I denotes the coexisting steady state of the healthy and leukaemia species and area II represents the healthy steady state. Negative Feedback enables differentiated blood cells *D* to signal to their parent population *A* and *S* and transmit information about their population size before leaving the bone marrow and entering the blood stream. This allows haematopoietic stem cells *S* and haematopoietic progenitor cells *A* to adjust their probability *p*_10_ and *p*_20_ of differentiation to fluctuating demands, and properly enhance the self-renewal ability of *S* and *A*. As a result, healthy progenitor cells are bestowed a competitive advantage over leukaemia stem cells [7].

## Discussion

The main goal of this paper is to qualitatively understand the pathogenesis of acute myeloid leukaemia. we employed a sophisticated mathematical model which includes some negative feedback inhibition from the terminally differentiated cells of haematopoietic lineages to the haematopoietic stem cells and haematopoietic progenitor cells to quantify the regulatory mechanisms mentioned above by a set of ordinary differential equations. We found that the weak negative feedback for differentiation probability is conducive to the cure of leukemia. However, with the strengthening of negative feedback, leukemia will be more difficult to be cured, and even induce death. In contrast, strong negative feedback for differentiation rate of progenitor cells can promote healthy haematopoiesis and suppress leukaemia.

In the modeling process, we mainly considered the competition between hematopoietic progenitor cells and leukemia stem cells in the microenvironment. In fact, this competitive relationship has also been confirmed by experiments at [5, 7]. However, one limitation of the current model is that we do not consider the time delay of substance delivery and non-uniform distribution of diseased cells, we hope to explore the role of time delay and heterogeneity in the pathogenesis of acute myeloid leukaemia in a future study.

## Conclusions

In recent decades, many scholars have focused on the specific pathogenesis of leukemia and have achieved some results [16–18]. There are many different ways to study the behaviour of a biological system. In this work, we mainly look at a mathematic model by bifurcation theory and numerical simulation technology. We have not only incorporate negative feedback mechanism on the existing model, but also constructed a model by using the modeling method of stem cell theory.

From the above sections, we can see that the weak negative feedback *g*_1_, *g*_2_, and *h*_1_ are conducive to the cure of leukemia. Part of these results are in consistent with the conclusions of [10]. However, with the strengthening of those negative feedback, leukemia will be difficult to be cured, and even induece death. In fact, negative feedback enables differentiated blood cells D to signal to their parent population A and S and transmit information about their population size before leaving the bone marrow and entering the blood stream. This allows haematopoietic stem cells S and haematopoietic progenitor cells A to adjust their probability *p*_10_ and *p*_20_ of differentiation to fluctuating demands, and properly enhance the self-renewal ability of S and A. In contrast, the strong negative feedback *h*_2_ can promote healthy haematopoiesis and suppress leukemia. This situation is in line with the conclusion of [7]. As a result, healthy progenitor cells are bestowed a competitive advantage over leukaemia stem cells. This paper is not only provides a perspective of bifurcation to investigate the pathogenesis of leukemia initiation theoretically, but also has practical implications for the therapy of leukemia and provides targets for therapy.
